# Revisiting the Concept of Targeting NFAT to Control T Cell Immunity and Autoimmune Diseases

**DOI:** 10.3389/fimmu.2018.02747

**Published:** 2018-11-27

**Authors:** Jae-Ung Lee, Li-Kyung Kim, Je-Min Choi

**Affiliations:** ^1^Department of Life Science, College of Natural Sciences, Hanyang University, Seoul, South Korea; ^2^Research Institute for Natural Sciences, Hanyang University, Seoul, South Korea

**Keywords:** NFAT, T cell, autoimmune disease, immune modulatory drugs, NFAT5

## Abstract

The nuclear factor of activated T cells (NFAT) family of transcription factors, which includes NFAT1, NFAT2, and NFAT4, are well-known to play important roles in T cell activation. Most of NFAT proteins are controlled by calcium influx upon T cell receptor and costimulatory signaling results increase of IL-2 and IL-2 receptor. NFAT3 however is not shown to be expressed in T cells and NFAT5 has not much highlighted in T cell functions yet. Recent studies demonstrate that the NFAT family proteins involve in function of lineage-specific transcription factors during differentiation of T helper 1 (Th1), Th2, Th17, regulatory T (Treg), and follicular helper T cells (Tfh). They have been studied to make physical interaction with the other transcription factors like GATA3 or Foxp3 and they also regulate Th cell signature gene expressions by direct binding on promotor region of target genes. From last decades, NFAT functions in T cells have been targeted to develop immune modulatory drugs for controlling T cell immunity in autoimmune diseases like cyclosporine A, FK506, etc. Due to their undesirable side defects, only limited application is available in human diseases. This review focuses on the recent advances in development of NFAT targeting drug as well as our understanding of each NFAT family protein in T cell biology. We also discuss updated detail molecular mechanism of NFAT functions in T cells, which would lead us to suggest an idea for developing specific NFAT inhibitors as a therapeutic drug for autoimmune diseases.

## Introduction: classical NFATs

Nuclear factor of activated T cells (NFAT) is a family of transcription factors identified in activated T cells, which promote the expression of interleukin-2 (IL-2) and the IL-2 receptor (1–3). Ligation of the T cell receptor (TCR) with antigen: major histocompatibility complex class II (MHCII) mediates multiple signaling cascades, including phospholipase C (PLC)-dependent pathways, which generates the secondary messengers inositol-1,4,5-triphosphate (IP_3_) and diacylglycerol (DAG). IP_3_ binds to IP_3_ receptor in the endoplasmic reticulum (ER) and releases Ca^2+^ ions to the cytoplasm (4, 5). Calmodulin captures free Ca^2+^ ions and activates the serine/threonine phosphatase calcineurin. Calcineurin dephosphorylates multiple serine residues in NFATs, resulting in their translocation into the nucleus (5, 6). NFAT proteins differentially regulate the expression of genes related to T cell development, activation, and differentiation (1, 7–11).

**Figure 1:**
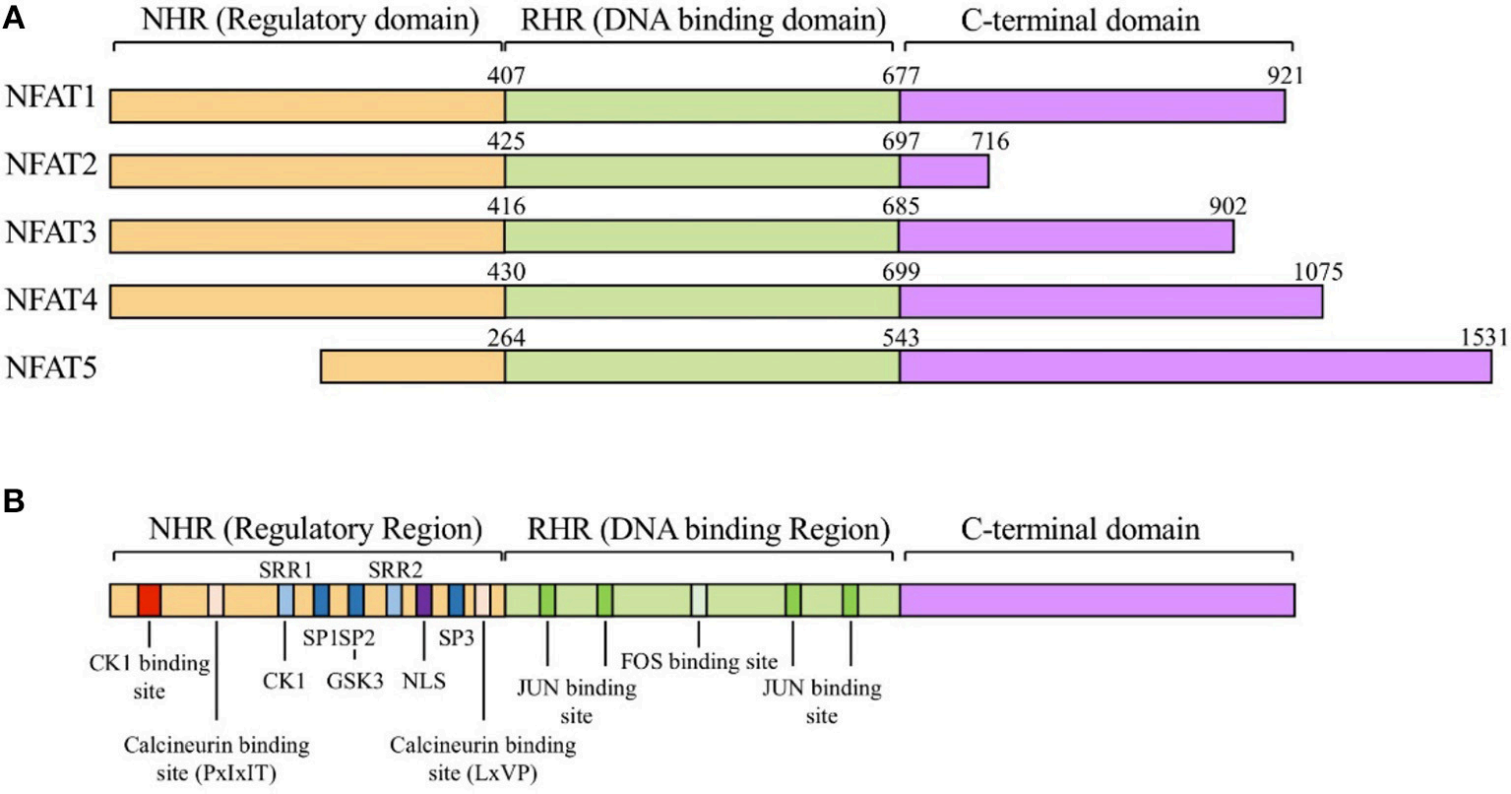
The NFAT protein family and structure of NFATs. **(A)** General structure of nuclear factor of activated T-cells (NFAT) protein family. The NFAT family consists of five protein members: NFAT1 (NFATc2 or NFATp), NFAT2 (NFATc1 or NFATc), NFAT3 (NFATc4), NFAT4 (NFATc3 or NFATx), and NFAT5 (TonEBP or OREBP). NFAT proteins contain a REL homology region (RHR), NFAT homology region (NHR), and C-terminal domain. RHR, which binds to DNA, is the most conserved domain, and NHR, the regulatory domain, is conserved from NFAT1 to NFAT4, but not in NFAT5. **(B)** Schematic alignment of NFAT proteins. NHR consists of several conserved regulatory motifs. The NHR contains casein kinase 1(CK1), GSK3 docking site, calcineurin docking site, and PxIxIT and LxVP motifs. NFAT5 lacks the NHR regulatory domain. NHR also contains a nuclear localization signal (NLS), which is required for nucleus translocation, serine-rich regions (SRRs), serine- proline-repeat motifs (SPs), and a phosphorylation site that is dephosphorylated by regulatory phosphatases such as calcineurin, CK1, and GSK3. RHR, the DNA binding domain, contains binding sites for FOS and JUN. NFAT5 lacks binding motifs for FOS and JUN.

The NFAT family proteins share a conserved N-terminal NFAT-homology region (NHR) and REL-homology region (RHR). The NHR is moderately conserved among NFAT family members and contains several serine-rich regions (SRR) and a transactivation domain. The NFAT family consists of five proteins: NFAT1 (NFATc2 or NFATp), NFAT2 (NFATc1 or NFATc), NFAT3 (NFATc4), NFAT4 (NFATc3 or NFATx), and NFAT5 (TonEBP or OREBP) (Figure 1) (12). NFAT1 is constitutively expressed in normal human T cells, whereas NFAT2 is induced by activation (13). NFAT1 and NFAT2 are regulated by calcineurin, which dephosphorylates NFATs and promotes their nuclear translocation (12). NFAT3 is rarely expressed in T cells because of lower chromatin accessibility and enhancer activity of its promoter (14). NFAT4 is weakly expressed in unstimulated cells and its expression is not enhanced by activation (13). NFAT1 and NFAT2 are the most-studied NFAT family members because of their high expression level in T cells. NFAT1 and NFAT2 surpass the ability of NFAT4 to bind to their target cytokine promoters (15). NFAT5 is expressed by almost all cells and is activated in response to osmotic stress (16). Translocated NFAT proteins interact with different transcription factors (such as AP1, FOXP3, and BATF) (1, 17–19). Depending on partner proteins, NFATs can either enhance immune responses or induce immune tolerance. AP1, the most widely known partner protein of NFATs, forms a complex with NFATs and induces various cytokines (such as IL-2, IL-4, and IFN-γ) and other T cell activation-induced proteins (7).

As NFATs are involved in diverse molecular interactions, they are tightly regulated by post-translational modifications in the normal state (12). Several kinases, including casein kinase 1 (CK1), glycogen synthase kinase 3 (GSK3), JUN N-terminal kinase (JNK), and p38, phosphorylate the serine-rich motifs located in the NHR domain of NFAT proteins and maintain them in inactive state (20–23). In addition to phosphorylation, NFAT can be regulated by protein acetylation, proteolytic cleavage, and SUMOylation by the small ubiquitin-like modifier (SUMO) (24–26).

Considering the important role of NFAT proteins in regulation of T cell activation, several therapeutic approaches were developed to inhibit NFAT signaling. Calcineurin inhibitors, such as cyclosporine A (CsA) and tacrolimus (FK506), have been used to treat graft rejection and autoimmune diseases, including atopic dermatitis, rheumatoid arthritis, and lupus nephritis (27–32). More inhibitors specifically targeting NFATs (such as VIVIT peptide, INCA-1, ST-1959, and UR-1505) were developed and are being verified; however, they are yet to be analyzed in suitable animal models of autoimmune diseases to investigate their potential of ameliorating diseases (33–36).

## NFAT in T cell subsets: Th1, Th2, Th17, treg, and Tfh

**Figure 2:**
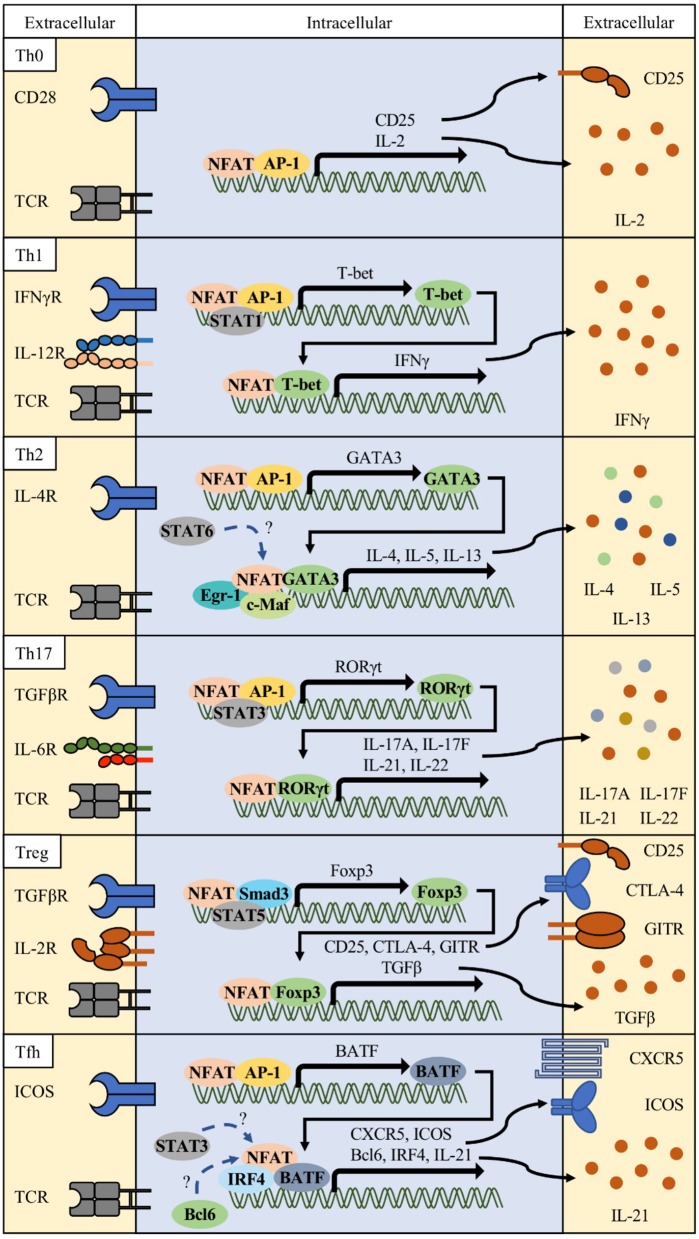
Various combinations of NFAT and interacting partner proteins in T helper cells. Differentiation of each Th cell is initiated by activation of T cell receptor (TCR), costimulatory receptor signals (e.g., CD28 or ICOS), and specific lineage determining cytokine signals. These signals orchestrate to induce the NFAT/AP-1 complex to express lineage-related transcription factors such as T-bet for Th1, GATA3 for Th2, RORγt for Th17, and BATF for follicular helper T (Tfh) cells. In combination with these transcription factors, NFAT/partner protein complexes determine their lineage differentiation and functional characteristics (surface receptors and cytokine production).

Th1: Although NFAT was originally identified to play important roles in the activation of T cells, it has also been shown that NFAT proteins differentially affect T helper (Th) cell differentiation (Figure 2) (37–41). Each differentiated T subset is characterized by the expression of their specific master regulator transcription factors and signature cytokines. Th1 cells are essential effector T cells against intracellular bacteria and virus infections (42, 43). Th1 differentiation is induced by TCR signaling and priming cytokines such as IFN-γ and IL-12 (44, 45). Together with antigen stimulation, cytokine-mediated signal transducer and activator of transcription 1 (STAT1) activates T-bet (TBX21), a master transcription factor of Th1 (46, 47). The expression of NFAT2a, an isotype of NFAT2, is more elevated in Th1 and Th2 than in Th17 and Treg (48). NFAT1 binds to IFN-γ promoter region (11, 49). Loss of NFAT1 promotes mild bias toward Th2 cell differentiation with decreased production of IFN-γ and increased production of IL-4 (38, 39, 44, 50–52). In double knock-out (DKO) mice, the levels of Th2-related cytokines such as IL-4 and IL-5 increased 25- to 75-fold compared to in wild type mice with increased IgG1 and IgE titers (39). Recent studies showed that Ca^2+^ response is more intense and sustained in Th1 and that NFAT nuclear localization is shorter in Th2 than in Th1 (53), suggesting that NFAT1 and/or NFAT4 are positive regulators of Th1 inflammation.

Th2: In contrast, *Nfat2*-deficient mice show impaired production of IL-4 and Th2 cytokines and reduced IgG1 and IgE levels (40). Th2 cells express IL-4, IL-5, and IL-13, which stimulates mucosal immunity against parasite infections (54–56). Various transcription factors, namely, GATA3, STAT6, RBPJκ, MAF, IRF4, and JUNB, have been implicated in Th2 differentiation and function (10, 57–61). GATA3 forms a chromatin hub with NFAT1 in *Il4* and *Il13* promoter regions (62). IRF4 synergizes with NFAT1 and c-Maf to augment *Il4* promoter activity (10, 40). Ubiquitin-specific peptidase 4 (USP4) interacts with IRF4 and NFAT1 to enhance NFAT-mediated *Il4* promoter activity (63). RUNX3 physically interacts with NFAT2 and suppresses IL-4 production (64). NFAT1 competitively binds to the *CRTh2* promoter with GATA3 and negatively regulates CRTh2 expression, which mediates the production of Th2 cytokines such as IL-4, IL-5, and IL-13 (65). *Nfat1* deficiency increased Th2 cytokine levels, enhanced chromatin accessibility, and increased DNA demethylation in the *Il4* promoter region, inducing preferential recruitment of JUNB/SATB1 to the *Il4* promoter (51, 52). Similarly, *Nfat1/4* DKO CD4 T cells secrete large amounts of IL-4 upon TCR stimulation, and show increased Th2 cytokine production, which is not dependent on IL-4 production (40). Early growth response protein-1 (EGR1) is expressed predominantly in Th2 and cooperatively binds to the *Il4* enhancer element with NFAT1/2 (66). IL-31 cytokine induction in Th2 cells require Ca^2+^ mediated NFAT1/2 activation (67). NFAT2 and STAT6 synergistically enhance *Il31* promoter activity. These studies suggest that NFAT2 plays positive regulatory roles in Th2 inflammation with possible reciprocal relationship with NFAT1 or NFAT4.

Th17: Th17 subsets are important players in protection against extracellular pathogens and inflammatory response in autoimmune diseases (68, 69). Signature cytokines including IL-17A, IL-17F, IL-21, and IL-22 produced by Th17 cells induce massive tissue reaction such as neutrophil recruitment (70). NFAT is also important in the induction of these cytokines. NFAT1 and 2 directly bind to the *Il17* promoter region (71–74). CD4-specific *Nfat2*-deficient mice showed reduced IL-17 expression, and *Nfat1* and *Nfat2*-deficient mice (DKO) showed reduction in IL-17 expression (75). In a model of experimental colitis, *Nfat1* deficiency showed protective effects with reduced production of IL-6 and IL-17 by mucosal T lymphocytes (76). Hyperactivation of NFAT1, increased affinity for calcineurin, and decreased affinity for CK1, resulted in higher IL-17 and IL-10 production because of direct binding of NFAT1 to distal regulatory regions of *Il17* and *Il10* loci (73). Although NFAT1 hyperactivation induced production of IL-17 *in vitro*, mice were more resistant to induction of experimental autoimmune encephalomyelitis (EAE), with increased production of IL-10 and accumulation of Treg cells in the central nervous system. Conversely, CD4-specific *Nfat2*-deficient mice showed reduced levels of RORγt, a master transcription regulator of Th17, as well as reduction in IL-17A, IL-17F, and IL-21 production and protected from EAE (77). Although *Nfat1*-deficient mice also showed decreased inflammatory response in the EAE model, the underlying mechanism is different from that in *Nfat2*-deficient mice. CD4-specific *Nfat1*-deficient T cells secrete IL-17 along with IL-4 and IL-10, and these non-pathogenic Th17 cells contribute to protection from diseases (78). The above observations suggest that both NFAT1 and NFAT2 contribute to Th17 response.

Treg: FOXP3^+^ Treg cells are a distinct population suppressing other effector Th cells (79) and is divided into thymus-derived natural FOXP3^+^ (nTreg) T cells and peripheral inducible Treg (iTreg) (80). Studies on Treg cells were facilitated by the identification of mutations in *Foxp3* in mice and patients of immunodysregulation polyendocrinopathy enteropathy X-linked (IPEX) syndrome (81–83). Treg-mediated immune suppression is caused by multiple mechanisms such as CTLA-4-, IL-10-, TGFβ-, and antigen presenting cell (APC)-mediated indirect inhibition (84–86). Most of these Treg-related molecules are regulated by NFAT proteins (17, 73, 87). Ablation of *Nfat1, Nfat2*, and *Nfat4* alone or in combination such as *Nfat1/2* and *Nfat1/4* double KO diminished iTreg but not nTreg differentiation, suggesting specific roles of the NFAT family in peripheral activation and differentiation of regulatory T cells from naïve T cells (75). Studies show that NFAT facilitates the interaction between conserved noncoding sequence 2 (CNS2) at the *Foxp3* locus and *Foxp3* promoter, and that NFAT2 directly regulates SMAD3 and FOXP3 binding to CNS1, enhancing production of effector molecules in Treg (88–91). Specific inhibition of NFAT1/FOXP3 interaction using a FOXP3-derived peptide, FOXP3 393–403, impaired Treg-mediated suppressor function in a dose-dependent manner (92). This peptide also inhibited Treg differentiation in mice and human T cells and showed enhanced antitumor responses. However, several recent studies have reported that *Nfat* KO mice show increased GITR^+^ Treg cells in the lung after allergen challenge and protection in graft-vs.-host diseases (GvHD) (93, 94). The functions of NFAT in Treg responses are still controversial and more accurate studies are required.

Tfh: Tfh cells were recently identified as helper T cells expressing transcription factor B-cell lymphoma 6 (BCL6) (95). Tfh cells are distinguished from other Th cells by their selective role in inducing germinal center (GC) responses, with promotion of antibody class switching, somatic hypermutation, high affinity antibody production, and plasma cell differentiation (96). Tfh cells express the C-X-C motif chemokine receptor 5 (CXCR5) and localize into the GC of draining lymph nodes (97). In addition to BCL6, other transcription factors including STATs, MAF, BATF, IRF4, ASCL2, LEF-1, and TCF-1 are also essential for Tfh differentiation and function (98–103). Both Tfh and GC B cells express high levels of NFAT1 and NFAT2, which is indicative of the functional importance of NFAT family in humoral immunity (104, 105). Increased humoral responses were observed in *Nfat*1- and *Nfat4*-deficient mice with increased serum levels of IgG1 and IgM (39). In contrast, *Nfat2*-deficient T cells show reduced IgG1 and IgE levels (106). Thereafter, some studies reported that NFAT regulates the expression of molecules important for Tfh cell function and differentiation, such as interferon regulatory factor 4 (IRF4), programmed death-1 (PD-1), and CXCR5 (10, 104, 107). It is also known that NFAT is required for IL-21 production, which is important for Tfh differentiation and function, and its production was ablated by CsA treatment (108–110). However, IL-21 inhibits Tfr expansion via a BCL6-mediated mechanism (109). NFAT2 has especially high expression in human and mouse Tfh cells, and NFAT1 level increased slightly in mouse Tfh cells (CD4^+^ICOS^+^CXCR5^+^) (104, 111), although NFAT3 and NFAT4 expression was not induced. Nuclear level of NFAT2a, an isoform of autoregulated NFAT2, and *Nfat2* P1 promoter activity increased in Tfh cells, which is suggestive of specific roles of NFAT2 in Tfh cells. T cell-specific *Nfat2*-deficient mice (*Nfat2*^*fl*/*fl*^ *x Cd4*^*cre*^) showed increase in Tfh cell population and GC responses in mesenteric lymph nodes and elevation in 4-hydroxy-3-nitrophenylacetyl (NP)-specific IgM, IgG1, and IgG3 levels after NP-conjugated keyhole limpet hemocyanin (KLH) immunization. We reasoned that the increase in Tfh population and GC responses in *Nfat2*-deficient mice was due to impaired expression of CXCR5 in follicular regulatory T cells (Tfr). In fact, *Nfat2* deletion in Treg cells (*Foxp3-IRES-Cre x Nfat2*^*fl*/*fl*^) reduced the Tfr population, and similar responses were observed with CD4-specific *Nfat2*-deficient mice upon immunization. However, in a model of lymphocytic choriomeningitis virus (LCMV) infection, *Nfat1/2*-deficient mice showed impaired Tfh differentiation with reduced production of LCMV-specific antibody and functional molecules such as PD-1, ICOS, Ly108, CXCR5, and SLAM (112). In this model system, adoptively transferred LCMV-specific CD4 T cells (SMARTA CD4 T cells) also showed decreased Tfh differentiation and GC responses. Abolished store-operated Ca^2+^ entry (SOCE), which is a driving mechanism of NFAT activation in T cells, exhibits spontaneous increase in GC B cells and humoral autoimmunity due to low Tfr induction, whereas LCMV infection reduced Tfh cell differentiation and LCMV-specific IgG titers, suggesting different roles of NFAT depending on specific antigenic stimulation and environment, including presence of other immune cell types (19). Thus, NFAT1 and NFAT2 appear to exert positive regulatory effects on Tfh differentiation or function, whereas NFAT3 and NFAT4 are not required for Tfh or humoral immunity.

## NFAT5 in T cells

NFAT5, also known as tonicity-responsive enhancer binding protein (TonEBP) or osmotic response element binding protein (OREBP), is the most recently identified member of the NFAT family (113–116). NFAT5 does not possess calcineurin binding domain, and is hence the only NFAT family protein that is not regulated by calcium signaling (117). In response to osmotic stress, NFAT5 is activated by p38/MAPK signaling and regulates the expression of osmoprotective genes required for normal function (118). Therefore, studies on NFAT5 initially focused primarily on kidney medulla, skin, and eyes exposed to hypertonicity (119). However, NFAT5 is expressed not only in these tissues but also in the thymus and activated T lymphocytes (16). NFAT5 binds to TNF-α and lymphotoxin β promoter, suggesting that NFAT5 plays another role in the immune system, especially in T cells (120). Dominant negative (DN) *Nfat5* transgenic mice presented impaired thymic development and reduced peripheral T cell numbers. In addition, transgenic T cells and Jurkat cell lines expressing DN *Nfat5* also exhibited impaired proliferation and viability (121). Furthermore, *Nfat5*-null mice had hypernatremia and T cell lymphopenia, whereas T cell-specific *Nfat5* knockout mice had isotonic plasma and normal T cell numbers, but decreased survival and proliferation in hypertonic condition. These altered T cell homeostasis are associated with NFAT5-dependent CD24 induction in T cells (122). Other studies have shown that NFAT5 also has osmostress-independent functions. In the thymus, NFAT5 regulates the progression from double-negative stage and therefore controls survival during thymocyte development. *Nfat5*-deficient mice had smaller thymus and less mature CD4 and CD8 cells in the spleen and lymph nodes (123). Recent studies showed that high salt conditions promote the differentiation of naïve T cells into Th17 cells via NFAT5-dependent mechanisms with more pathogenic characteristics and GM-CSF production. Short hairpin RNA (shRNA)-mediated silencing of *Nfat5* in CD4 T cells decreased IL-17A and CCR6 expression in Th17 polarizing conditions, suggesting a new role in the pathogenesis of autoimmune diseases involving NFAT5 activation (124, 125). High-salt diet increases the number of Th17 cells *in vivo* and aggravates EAE via the NFAT5/SGK1 pathway (125). Under hypertonic conditions, NFAT5 enhances the expression of the pathogenic Th17–related cytokine IL-17A and Th17-assosiated genes, *Rorc* and *Il23r*, in T cells. In contrast, *in vivo*-activated *Nfat*-deficient CD4 T cells were skewed toward increased IFNγ and IL-17A expression, and T cell-restricted *Nfat5*-deficient mice exhibited more severe pathology and enhanced IFNγ mRNA expression in lymph nodes and colon of an animal model of experimental colitis (126). Recent studies identified that several miRNAs can target *Nfat5*. miR-20b was studied in thymoma-associated myasthenia gravis, where it inhibited NFAT5 expression with reduced T cell proliferation (127). miR-568 expression decreased during Treg activation and correlated inversely with NFAT5 expression. Overexpression of miR-568 inhibited Treg differentiation and TGFβ and IL-10 production (128). Another study showed that the expression of the microRNA cluster 106a~363 decreased during Th17 cell differentiation and that over-expression of miR-18b, miR-106a, and miR-363-3p reduced Th17 differentiation (129). These effects of the miRNA 106a~363 cluster are mediated by inhibition of their targets such as *Nfat5* and *Rorc*. Patients with inflammatory bowel disease (IBD), including those with Crohn's disease (CD), ulcerative colitis (UC), and autoimmune enteropathy (AIE), have significantly reduced NFAT5 expression (130). NFAT5 inhibition in healthy human and murine T cells exhibited disruption in cytokine production and survival (130).

We have briefly summarized the role of each NFAT family proteins in T cell immunity (Table 1). These observations suggest that NFAT5 plays an important role in T cells under both hypertonic and isotonic conditions and in Treg function. NFAT5 may be a new attractive target for the treatment of autoimmune diseases irrespective of calcium-mediated adverse effects.

**Table 1:** NFAT family in T cell immunity.

**Regulation**	**NFAT family member**	**Expression in the immune system**	**Functions in T cell immunity**
Ca^2+^/Calcineurin	NFAT1	Expressed in all types of Th cells	Positive regulator of Th1 inflammation. Positively regulate Th17-cytokines, IL-17, and IL-6. Interacts with FOXP3 and enhances effector molecules in Treg.
	NFAT2	Expressed in all types of Th cells	Positive regulatory role in Th2 responses Stimulates RORγT and Th17-realted cytokines Positively regulates Treg differentiation with NFAT1/4 Enhances Tfh differentiation and function
	NFAT3	Rarely expressed in T cells	Unknown
	NFAT4	Expressed in thymocytes and weakly expressed in peripheral T cells	Thymocyte development and survival TCR hyper-reactivity Positive synergy with NFAT1 in Th1 and Treg response
Osmotic stress	NFAT5	Expressed in thymocytes and iTreg	Thymocyte development and survival Hypersensitivity in hypertonic condition Th17-mediated disease pathogenicity

## NFAT targeting drugs for autoimmunity: beyond cyclosporine a and tacrolimus

Considering the important role of NFAT signaling in T cell function, NFAT has long been considered as an attractive target for therapeutic approaches to control autoimmune responses and graft rejection (Table 2) (30, 31, 163, 164). The most well-known drugs targeting NFAT are the calcineurin inhibitors CsA and FK506. CsA was first identified in 1971 from the fungus *Tolypocladium inflatum* (165, 166). Later, tacrolimus, also known as FK506, was isolated from a fungus named *Streptomyces tsukubaensis* (167). CsA and FK506 function similarly in that they bind to immunophilins called cyclophilin and FK-binding protein 12 (FKBP12), respectively (168–170). This inhibitor-immunophilin complex directly binds to calcineurin and inhibits its phosphatase activity, thereby inhibiting NFAT dephosphorylation (171). Both drugs have been well-used to treat graft rejection and autoimmune diseases. CsA and tacrolimus are used in atopic dermatitis and in other autoimmune diseases, including lupus nephritis, and many clinical trials have been conducted to determine the efficacy of calcineurin inhibitors (30–32). In fact, calcineurin inhibitors positively affect the treatment of autoimmune membranous nephropathy (172, 173). Similarly, recent studies revealed that CsA inhibits Th17 cells in patients with Sjögren's syndrome and rheumatoid arthritis (174, 175). Treatment of rheumatoid arthritis with tacrolimus and other drugs such as methotrexate showed promising synergy in clinical results (27, 176). Although calcineurin inhibitors are effective in autoimmune disease therapy with inhibition of T cell activation, inhibition of calcineurin has serious drawbacks; for example, blocking of calcineurin phosphatase activity affects numerous targets of calcineurin as well as NFATs. Neurotoxicity and nephrotoxicity are the most common side effects of calcineurin inhibitors (177, 178). Calcineurin is also highly expressed in neural tissues (179). It regulates IP_3_ and the ryanodine receptor and thereby controls calcium flux in the cerebellum (180). It is also associated with gamma aminobutyric acid (GABA_A_) and N-methyl D-aspartate (NMDA) receptors (181, 182). Calcineurin also plays an important role in exocytosis and vesicle recycling of neurotransmitters and nitric oxide synthase (NOS) (183–185). Nephrotoxicity of calcineurin inhibitors is associated with TGFβ and endothelin production. TGFβ increases the extracellular matrix (ECM) by inducing collagen and fibronectin, resulting in tubular fibrosis and anti–TGFβ antibody-neutralized nephrotoxic effects (186, 187). Endothelin level is also increased by calcineurin inhibitors. Endothelin is related to endothelial dysfunction, impaired glomerular filtration, and systemic hypertension (188, 189). Recent studies have indicated that calcineurin inhibitors have a negative effect on regulatory T cell proliferation and function, which are necessary for immune tolerance (190, 191). Hence, investigations for identifying more selective and less toxic inhibitors without affecting calcineurin activity are underway.

**Table 2:** Calcineurin-NFAT inhibitors and their mechanisms.

**Inhibitors**	**Mechanism**	**Inhibitory effect**	**References**
**INHIBITORS THAT INHIBIT CALCINEURIN ACTIVITY**			
Cyclosporine A	Binds with cyclophilin and inhibits calcineurin activity.	Inhibits T cell proliferation and cytokine expression.	(131–133)
Tacrolimus	Binds with FKBP12 and inhibits calcineurin activity.	Inhibits T cell proliferation and cytokine expression.	(134–136)
Voclsporin (ISA247)	Binds with cyclophilin and inhibits calcineurin activity.	Better efficacy than cyclosporine A.	(137)
Pimecrolimus	Binds with FKBP12 and inhibits calcineurin activity.	Inhibits T cell proliferation and cytokine expression.	(138)
Thiopental	Binds to calcineurin and inhibits calcineurin activity.	Inhibits T cell proliferation and IL-2, and IFNγ expression	(139)
Kaempferol	Binds to the catalytic domain of calcineurin A and inhibits calcineurin activity.	Inhibits IL-2 expression in Jurkat cells.	(140, 141)
Tropisetron	Inhibits calcineurin activity.	Inhibits IL-2 production in primary T cells.	(142)
**INHIBITORS THAT INHIBIT CALCINEURIN-NFAT INTERACTION**			
PxIxIT peptide	Calcineurin docking site of NFAT; Inhibits calcineurin-NFAT binding.	Inhibits NFAT-dependent expression in Jurkat cells.	(143)
VIVIT peptide	Inhibits calcineurin-NFAT binding.	Inhibits IL-2 production and proliferation of Jurkat cells; Increases graft survival in islet transplantation mice.	(144, 145)
LxVP peptide	Inhibits calcineurin-NFAT binding and inhibits calcineurin activity.	Inhibits IL-2 production in Jurkat cells.	(146)
AKAP79	Inhibits calcineurin-NFAT binding.	Inhibits IL-2 production in T cells.	(147)
Cabin-1/Cain	Inhibits calcineurin-NFAT binding.	Inhibits IL-2 promoter activation in T cells.	(148)
INCA-1,2, and 6	Inhibits calcineurin-NFAT binding.	Inhibits cytokine expression in T cells.	(34)
Dipyridamole	Inhibits calcineurin-NFAT binding.	Inhibits cytokine production.	(149)
NCI3	Causes allosteric changes in calcineurin and inhibits calcineurin-NFAT binding.	Inhibits T cell proliferation and IL-2 expression in Jurkat and primary human T cells.	(150)
**INHIBITORS THAT AFFECT NFAT MIGRATION**			
ST1959	Induces NFAT1 nuclear export.	Inhibits T cell activation, proliferation, and cytokine production.	(35)
Helenalin	Inhibits NFAT1 nuclear translocation.	Inhibits T cell proliferation and IL-2 production.	(151)
Roc-1,2, and 3	Inhibits NFAT2 nuclear translocation.	Inhibits IL-2, IL-4, and IFNγ expression.	(152)
**INHIBITORS THAT DIRECTLY AFFECT NFAT STABILITY**			
Zoledronic acid	Induces NFAT degradation by inhibition of GSK3β	Inhibits cell growth by inducing G1 cell cycle arrest.	(153)
Genistein	Reduces mRNA and protein expression of NFAT.	Induces apoptosis; decreases number of T cell.	(154)
**INHIBITORS THAT INHIBIT NFAT-DNA INTERACTION**			
UR-1505	Inhibits NFAT binding to DNA.	Inhibits T cell proliferation and IFNγ expression.	(155)
Caffeic acid phenethyl ester (CAPE)	Inhibits NFAT nuclear translocation and DNA binding.	Inhibits proliferation and IL-2 production o f T cells.	(156)
Punicalagin	Inhibits NFAT nuclear translocation and DNA binding.	Inhibits IL-2 production of CD4+ T cells.	(157)
Imperatorin	Inhibits NFAT nuclear translocation and DNA binding.	Inhibits T cell proliferation.	(158)
WIN 53071	Alters NFATc-DNA complex formation.	Inhibits IL- 2 expression in primary human T cells.	(159)
YM-53792	Inhibits NFAT1-DNA binding.	Inhibits IL- 2, IL-4 expression in primary human T cells.	(160)
AM-404	Inhibits NFAT1-DNA binding.	Inhibits T cell proliferation and IL-2 and TNFα transcription.	(161)
Digitoxin	Inhibits NFAT1 binding to c-Myc promoter.	Inhibits proliferation and induces apoptosis.	(162)
**INHIBITORS THAT INHIBIT NFAT-TRANSCRIPTION PARTNER INTERACTION**			
FOXP3 393-403	Inhibits FOXP3-NFAT binding	Inhibits conversion into regulatory cells and enhances T cell proliferation.	(92)

To identify alternative NFAT inhibitors, a VIVIT peptide derived from the calcineurin-NFAT binding motif, PxIxIT, was developed to block NFAT binding to calcineurin and NFAT-dependent gene expression without affecting calcineurin phosphatase activity (143, 144). To resolve the delivery limitation of the VIVIT peptide, several studies modified VIVIT peptides using cell penetrating peptides (CPPs). 11R-conjugated VIVIT successfully increased transplant survival in islet transplanted mice (145). Other studies showed that Sim-2-conjugated VIVIT was efficiently delivered into cells and inhibited IL-2 and alleviated ovalbumin (OVA)-induced asthma in a murine model (192). In addition, the C-terminus of the regulatory domain possesses a conserved calcineurin binding motif, LxVP, which facilitates calcineurin docking and NFAT dephosphorylation (193, 194). However, LxVP presented weak binding strength for NFAT1 and affected calcineurin phosphatase activity (146, 195). Endogenous calcineurin inhibitors such as AKAP79, Cabin-1/Cain, MCIP1, and A238L have sequences similar to that of the PxIxIT motif (147, 148, 196–198).

Small molecules are similar in structure and function to classical inhibitors but have lesser side effects. Voclosporin (ISA247), an analog of CsA, possesses higher affinity to cyclophilin than CsA and was effective at lower concentrations (137). Therefore, it is considered a promising treatment option for arthritis and psoriasis (199, 200). Other drugs such as ST-1959, and Roc-1, 2, and 3 inhibit T cell responses by enhancing nuclear export of NFAT1 and NFAT2 (35, 152). Drugs such as zoledronic acid induce NFAT1 degradation via GSK3β inhibition (153). Certain inhibitors such as UR-1505 and digitoxin block the binding of NFAT to DNA (155). Remarkably, digitoxin specifically inhibits interaction between NFAT1 and the c-*Myc* promoter and thereby inhibits c-Myc-dependent transcription (162). The FOXP3-derived peptide, FOXP3 393–403, specifically inhibits FOXP3/NFAT interaction. This inhibitory peptide suppresses T cell conversion into iTregs and enhances T cell proliferation, thereby exhibiting antitumor effects (92). These strategies indicated that blockage of NFAT binding to a specific promoter or inhibition of its interaction to a particular transcriptional partner might selectively suppress its function.

To develop these NFAT inhibitory molecules as a new drug for human diseases, both T cells and other cells also should be considered for therapeutic purposes. Recent studies in myeloid cells have revealed the importance of NFAT in both innate and adaptive immunity. In an early response to pathogens, pattern recognition receptors (PRRs) such as TLR4 and dectin-1 induce the production of IL-2 from dendritic cells (201, 202). These signals activate PLCγ2 and promote NFAT-dependent IL-2 expression. In macrophages that express various NFAT family members except NFAT3, calcineurin/NFAT inhibitor treatment results in macrophages that are tolerant to lethal dose of lipopolysaccharide (LPS) (203–205). Other myeloid cells such as mast cells and neutrophils are influenced by Ca^2+^/NFAT signaling and produce cytokines and multiple immune mediators (206, 207). Therefore, NFAT targeting strategies should consider non-T cell mediated adverse effects as well as its potent effect of disease control and immune suppression.

Considering the multiple roles of calcineurin-NFAT signaling in both immune and non-immune cells, new methods for targeting NFAT are required. For peptide inhibitors such as VIVIT and LxVP, improved CPPs such as dNP2 can be used to enhance efficiency of *in vivo* delivery (208). Alternatively, more specific inhibition strategies other than calcineurin targeting can be used. Recent results regarding the role of each NFAT family member in T cells and the molecular mechanisms via which they regulate T cell responses indicate that new inhibitors that can block specific molecular interactions should be developed to reduce side effects and reinforce the efficacy of autoimmune disease therapy.

## Concluding remarks and perspectives

In the current review, we have summarized recent advances in our understanding of the role of NFAT family members in T cell responses and presented an overview of therapeutic agents targeting NFAT proteins for treating autoimmune diseases. Classically, NFAT has been studied as an important transcription factor for T cell activation under calcium signaling. However, recent studies revealed that NFAT function is not just limited to T cell activation but it also actively functions in differentiation of effector T cell subsets such as Th1, Th2, Th17, Treg, and Tfh cells. Based on better understanding of molecular mechanism of NFAT by direct interaction with T-bet, GATA3, RORγt, FOXP3, and BCL6, or by promoter binding to control T cell differentiation-related genes, we now are able to suggest a strategy to develop specific NFAT inhibitor to control a particular function of NFATs. Unlike other calcineurin-dependent NFAT proteins, NFAT5 in T cells is just recently recognized that it seems to be involved in thymocyte development and T cell survival and proliferation. Interestingly, it could be activated under high salt condition in T cells to commit more pathogenic Th17 differentiation in multiple sclerosis model. While it is still questionable whether specific NFAT5 inhibition in T cells would be beneficial for autoimmunity, it could be worth to investigate as a new target of NFAT inhibition for treating autoimmune diseases. As previously developed NFAT targeting drugs show significant adverse effects owing to the diverse calcium signaling-related target genes of NFAT proteins, a novel strategy either targeting specific NFAT family members or molecular interference of NFAT binding proteins will be more beneficial for controlling T cell function and autoimmune diseases.

## Author contributions

All authors listed have made a substantial, direct and intellectual contribution to the work, and approved it for publication.

### Conflict of interest statement

The authors declare that the research was conducted in the absence of any commercial or financial relationships that could be construed as a potential conflict of interest.
